# Looks like a surgeon? Impact of anthropometry on the kinematics of surgical novices: counterbalanced cross-over design study of minimally invasive surgery

**DOI:** 10.1007/s11701-026-04077-y

**Published:** 2026-10-26

**Authors:** Anumithra Amirthanayagam, Massimiliano Zecca, Anisha Chitrakar, Vijay Chavda, Esther L. Moss

**Affiliations:** 1 College of Life Sciences, University of Leicester, University Road, Leicester, LE1 7RH UK; 2 Wolfson School of Mechanical, Electrical and Manufacturing Engineering, Loughborough University, Loughborough, LE11 3TU UK; 3 Department of Gynaecological Oncology, University Hospitals of Leicester NHS Trust, Gwendolen Rd, Leicester, LE5 4PW UK

**Keywords:** Anthropometry, Musculoskeletal injury, Minimally invasive surgery, Laparoscopic, Robotic assisted, Kinematics

## Abstract

To investigate the relationship between anthropometric measurements of surgical novices and their upper-body kinematics during laparoscopic (LAP) and robotic assisted (RA) tasks. A counterbalanced crossover design study was conducted. Participants were assigned by systematic alternating allocation to first perform tasks on an Inovus laparoscopic trainer (LAP) or da Vinci Xi system (RA) in a simulation suite, whilst wearing three motion analysis sensors (upper left and right arms, and mid thoracic). In total 57 surgical novices (33 female, 24 male) were recruited. Kinematic data were available for 54 participants. Height, elbow height and arm span of female participants were significantly lower than male participants, 165.6 ± 5.42 cm, 107.9 ± 4.9 cm and 163.2 ± 10.2 cm versus 181 ± 8.3 cm 118.3 ± 6.6 cm and 182.7 ± 10.3 cm respectively (*p* < 0.0001). Greater jerk and angular velocity were demonstrated for LAP versus RA tasks (*p* < 0.0001). Significant interactions between anthropometry and surgical modality were observed; decreasing elbow height correlated with significantly greater angular velocity during LAP tasks (interaction p < 0.001; jerk *p* = 0.005), whereas no such relationship was observed for RA tasks. The interactions for height did not remain significant after correction for multiple comparisons. There is an inverse relationship between anthropometric measurements (in particular elbow height) and angular velocity recorded during simulated LAP, but not RA, tasks. Surgical novices at the lower end of the anthropometric spectrum exhibit significantly greater angular velocity and jerk when performing LAP compared to RA tasks. These kinematic differences provide a plausible mechanistic hypothesis for the higher rates of work-related musculoskeletal injuries reported in laparoscopy, requiring future longitudinal investigation.

## Introduction

Minimally invasive surgery (MIS) has become firmly incorporated into clinical practice over the past three decades due to improved surgical outcomes, including lower post-operative morbidity and mortality rates, and shorter recovery times [1–5]. However, the introduction of MIS has been associated with a reported increase in work-related musculoskeletal injuries (WMSI) sustained by surgeons [6, 7]. Common sites of injury include the upper and lower back, and shoulders [6, 7]. Symptoms have been reported to be exacerbated by patient factors, in particular body mass index [8], and surgeon factors, including anthropometry [9, 10], number of years in training and exposure to the surgical modality of choice, and surgical instrument design [11]. Current evidence shows that surgeon height can increase the risk of upper limb injuries [9, 10], particularly in female surgeons who are often of shorter height [10, 12, 13]. Systematic review of existing literature has demonstrated that the sex of the surgeon has a significant role in musculoskeletal symptoms, 59%-100% of female surgeons report issues compared to only 13% to 56% of male surgeons [14]. However, whether sex or anthropometry is the causative factor remains to be clarified.

Surveys have highlighted that the regular exposure to poor posture, creating greater muscle activation, along with high case volumes have led to cumulative work-related musculoskeletal injury [12, 15]. Conventional laparoscopy (LAP) has been shown to place greater strain on all muscle groups compared to robotic assisted (RA) procedures, demonstrated by persistently greater muscle activation in LAP, regardless of whether participants are experts or novices [15]. Hence an understanding of anthropometry, its influence on kinematics during MIS tasks and optimise the ergonomic use of MIS equipment, is vital to reducing long term injury risk [16].

The primary aim of this study was to examine the hypothesis that anthropometry directly correlates with kinematics, when performing MIS. To achieve this aim, the study was designed to quantify the impact of anthropometry on the kinematics of surgical novices performing tasks across two minimally invasive platforms (LAP and RA).

## Materials and methods

The study was conducted in accordance with the Declaration of Helsinki and approved by the Ethics Committee of University of Leicester (protocol code 3169) and was a sub-study of the ISSUE: Impact of Surgeon on The Surgeon study (ISRCTN11807724) which was funded by an Intuitive Surgical Ltd clinical research grant. The study design was a counterbalanced crossover design study with all participants undertaking three tasks LAP and RA in a simulated environment. The study was conducted over a 5-day period (3 full days and two half days) in February 2024. The da Vinci robotic system was loaned to the research team for 5 days to conduct the study. The RA tasks were performed on a demonstration model of the da Vinci Xi (Intuitive Surgical Ltd, USA) and the LAP tasks on the Inovus LAP trainer (Inovus Medical, UK). The motion capture equipment used was the Movella DOT sensor (Movella DOT, USA), which contains a 3-axis accelerometer (± 16 g), a 3-axis gyroscope (± 2000 °/s) and a 3-axis magnetometer (± 8 Gauss).

Medical students from the University of Leicester (all year groups) were invited to participate in the study. Recruitment was via email and supported by University’s administration team and surgical societies. Recruited participants self-selected a time/date to attend for testing. Prior to attendance, all participants were required to watch an information video detailing the positioning of the monitoring equipment and demonstrations of the exercises to be undertaken. Information was also included on how to use both the LAP equipment and robotic console, along with the console safety introductory video (Intuitive Surgical Ltd).

When attending for testing, information was collected on participants’ sex and anthropometric measurements, including height, arm span, elbow height, dominant hand, and hand size, before fitting three Movella DOT motion sensors, using adjustable elastic straps, on the left and right upper arms (mid humerus) and mid thorax. Participants were allocated to LAP or RA as the first modality by alternating the starting modality by day of testing, for example participants attending on Day 1 started LAP while those attending on Day 2 started RA. As participants self-selected their testing date, testing day and starting modality were linked. The order of the 3 tasks was varied by the supervising surgeons, with prospective monitoring to balance task order across modalities; no formal randomisation procedure was used. Each participant was required to perform three tasks: (1) placing rings on spikes; (2) stacking of cubes; and (3) moving a hoop along a fixed twisted wire. Each task was 5 min in duration (timed) with a 1–2-minute break in-between tasks. There was a 3–5-minute break between completing one modality and moving to the next. No task-performance measure (accuracy, repetitions or errors) was recorded.

For the LAP tasks the setup closely reflected the positioning observed in pelvic/gynaecological surgery. The laparoscopic box trainer was placed on an operating table set at 72 cm. The table height remained fixed throughout the study and was not altered for any participant. The participants were asked to stand to the left of the box trainer (at the head of the table) with the instruments pointing towards the foot of the table. The laptop monitor was placed on the operating table, in front of the box trainer and the supervising surgeon ensured the screen was visible for all participants to undertake the three tasks. For the RA tasks, the console was separate from the operating table where the tasks were set up, and participants were allowed to change the height of the RA console. Console settings were not recorded.

The primary outcome measurement was the relationship between the motion during each task (mean jerk and mean angular velocity) and participant height, measured by the upper-arm sensors. Secondary outcomes included the relationship between arm span, elbow height and hand size and the same measures. Other extracted features and the trunk sensor were exploratory.

### Motion data analysis

Data were acquired at 30 Hz and the general setting for recording of moderately dynamic movements selected on all sensors. Recorded data include sensor orientation, angular velocity and acceleration. Some recordings were lost because of sensor battery failure or data corruption; all available recordings were analysed (643 upper-arm and 283 trunk observations from 54 participants), and the mixed-effects models accommodate this imbalance without excluding participants with incomplete data. Data were transferred to a University of Leicester managed laptop using the Movella DOT app and imported. Availability of Euler and Quaternion data was checked as well as orientation, acceleration and gyroscope data. Minor differences in the length of the data due to sequential termination of recording of the sensors were corrected. Data clean-up was conducted with the removal of spurious spikes for gyroscope and acceleration data and adjustments for discontinuities of Euler data. Pre-processing included removal of artefacts and two sets of filtering. Firstly, low pass filtering (moving average), feature extraction (max, min, mean, root mean square (RMS), range, and standard deviation for all parameters), and normalisation was conducted individually for each measure analysed. High frequency noise was removed. Data were converted from the sensor reference frame to the real-world reference frame to remove the effect of gravity and rotated back to sensor reference frame to analyse task specific data only. The norm of each signal was computed, and the following features were extracted from it: maximum, minimum, mean, root mean square (RMS), range, standard deviation and spectral arc length (a measure of movement smoothness).

### Statistical analysis

All data processing and statistical analyses were performed using Python (version 3.12.8) with the *pandas* (version 2.2.3), *scipy* (version 1.15.2), and *statsmodels* (version 0.14.4) libraries. To appropriately account for the repeated-measures structure of the experimental design, in which each participant completed multiple simulated surgical tasks across both conventional LAP and RA modalities, Linear Mixed-Effects Models (LMM) were fitted using the *mixedlm* function from the *statsmodels.formula.api* module, rather than standard Ordinary Least Squares (OLS) regression or independent samples t-tests. For each kinematic outcome variable (e.g., angular velocity, jerk, and acceleration), a separate LMM was fitted. The primary analysis used the left and right upper-arm sensors; the trunk sensor was analysed separately. The model mathematically partitioned variance into fixed effects and random effects to resolve within-participant dependence. The formal model equation was defined as:

1 $$ \begin{aligned}Yijk & = \beta 0 + \beta 1Xi + \beta 2Mj + \beta 3\left( {Xi \times Mj} \right) \\& \quad + {\mathrm{Z}}\gamma + ui + vijk + \epsilon ijk \end{aligned}$$

Where:

- *Yijk* represents the kinematic measurement for participant *i*, using modality *j*, performing task *k*.
- *Xi* is the continuous anthropometric trait evaluated (e.g., height, elbow height, hand size, or arm span). To prevent multicollinearity, each trait was modelled independently.
- *Mj* is the categorical surgical modality (LAP vs. RA).
- *β3* represents the critical interaction term coefficient, explicitly capturing the differential impact of anthropometry depending on the surgical interface.
- Zγ is a vector of fixed-effect covariates controlling for participant sex, the specific simulated task (rings on spikes, stacking cubes and hoop along a twisted wire), potential sequence/period learning biases (starting modality and task order), and arm (dominant or non-dominant).
- ui ∼ N(0, σ2u) is the random intercept assigned to each participant i, accounting for intrinsic baseline differences in individual movement.

- vijk ∼ N(0, σ2v) is a random intercept for each participant-modality-task bout, shared by the two arm sensors recorded during the same task.
- *ϵijk* ∼ N(0, σ2) is the residual error.

Model parameters were estimated using Maximum Likelihood Estimation (MLE). Statistical significance was defined at an alpha level of 0.05. Where applicable, coefficients are reported alongside their 95% confidence intervals (CIs) to indicate the magnitude and direction of the effects. Slopes within each modality were derived from the fitted coefficients. Holm’s method [17, 18] was used to correct the eight anthropometry-by-modality interaction tests (four traits, two primary outcomes) for multiple comparisons. A carry-over effect was tested by adding a modality-by-starting-modality interaction, and sensitivity analyses used log-transformed outcomes, a participant random intercept only, and all three sensors. Model assumptions were checked by inspecting the residual distributions, which were approximately symmetric (skewness 0.3).

## Results

### Distribution of participant anthropometry

**Fig. 1:**
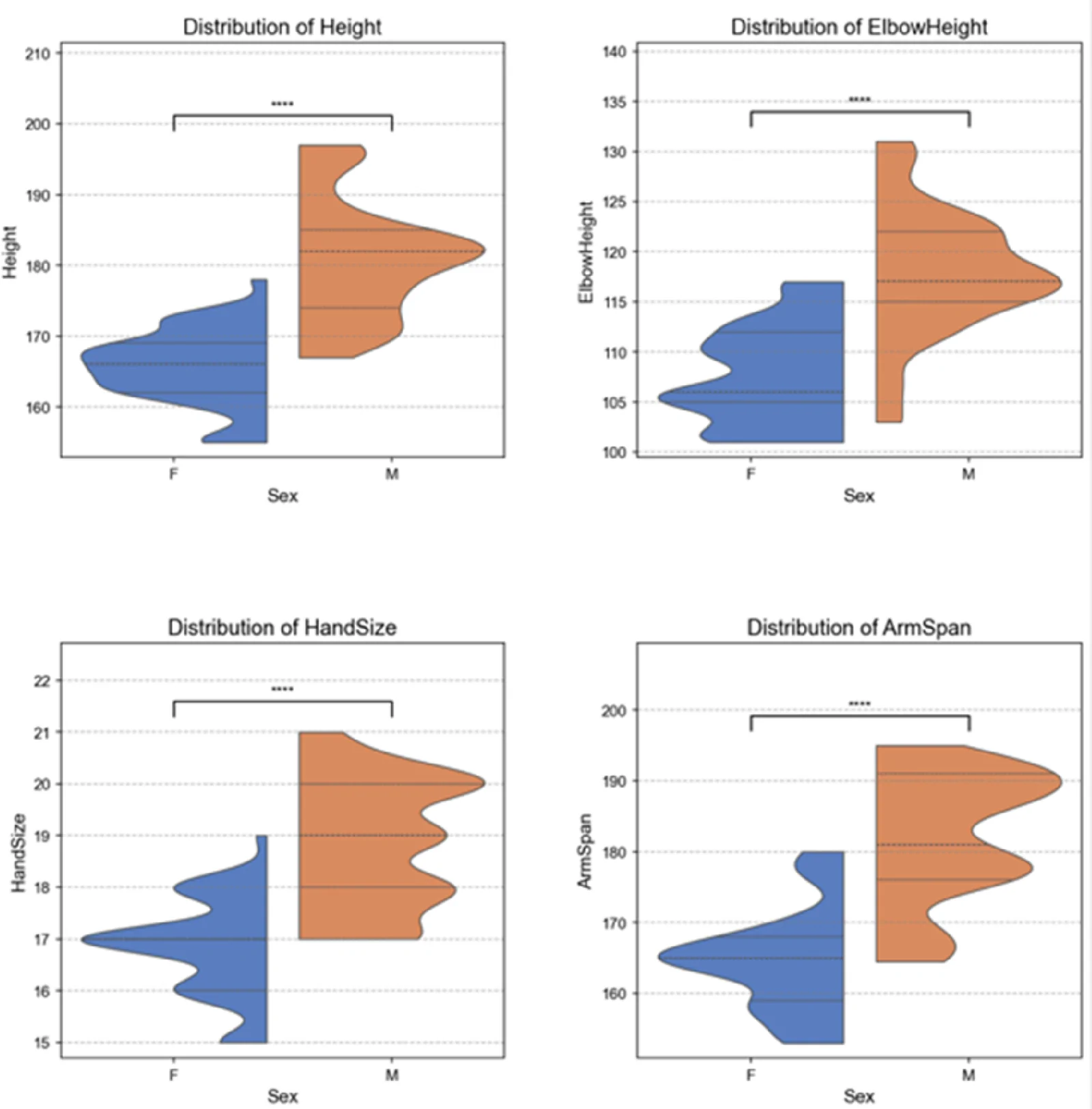
The distribution of the anthropometric features amongst the recruited participants *n* = 33 for females (F) and *n* = 24 for males (M).

In total, there were 57 participants, 33 females and 24 males. Kinematic data were available for 54 participants (31 females, 23 males). The male students had significantly greater anthropometric measurements as compared to the female participants (Fig. 1): mean height 181.1 ± 8.30 cm versus 165.6 ± 5.42 cm (*p* < 0.0001); mean arm span 182.7 ± 10.33 cm versus 163.2 ± 10.2 cm (*p* < 0.0001); mean elbow height 118.3 ± 6.58 cm versus 107.9 ± 4.9 cm (*p* < 0.0001); and mean hand size 18.88 ± 1.30 cm versus 16.89 ± 0.96 cm (*p* < 0.0001). Twenty-eight participants commenced with LAP tasks, and 29 RA tasks.

### Kinematic analysis

Parameters, including jerk and angular velocity, were analysed from the raw data. The right and left sensor data were used to capture the influence of the key anthropometric features (height, arm span, elbow height and hand size/dominance) on arm movements, whilst the centre sensor focused on the movements of the trunk.

#### LAP versus RA tasks

**Fig. 2:**
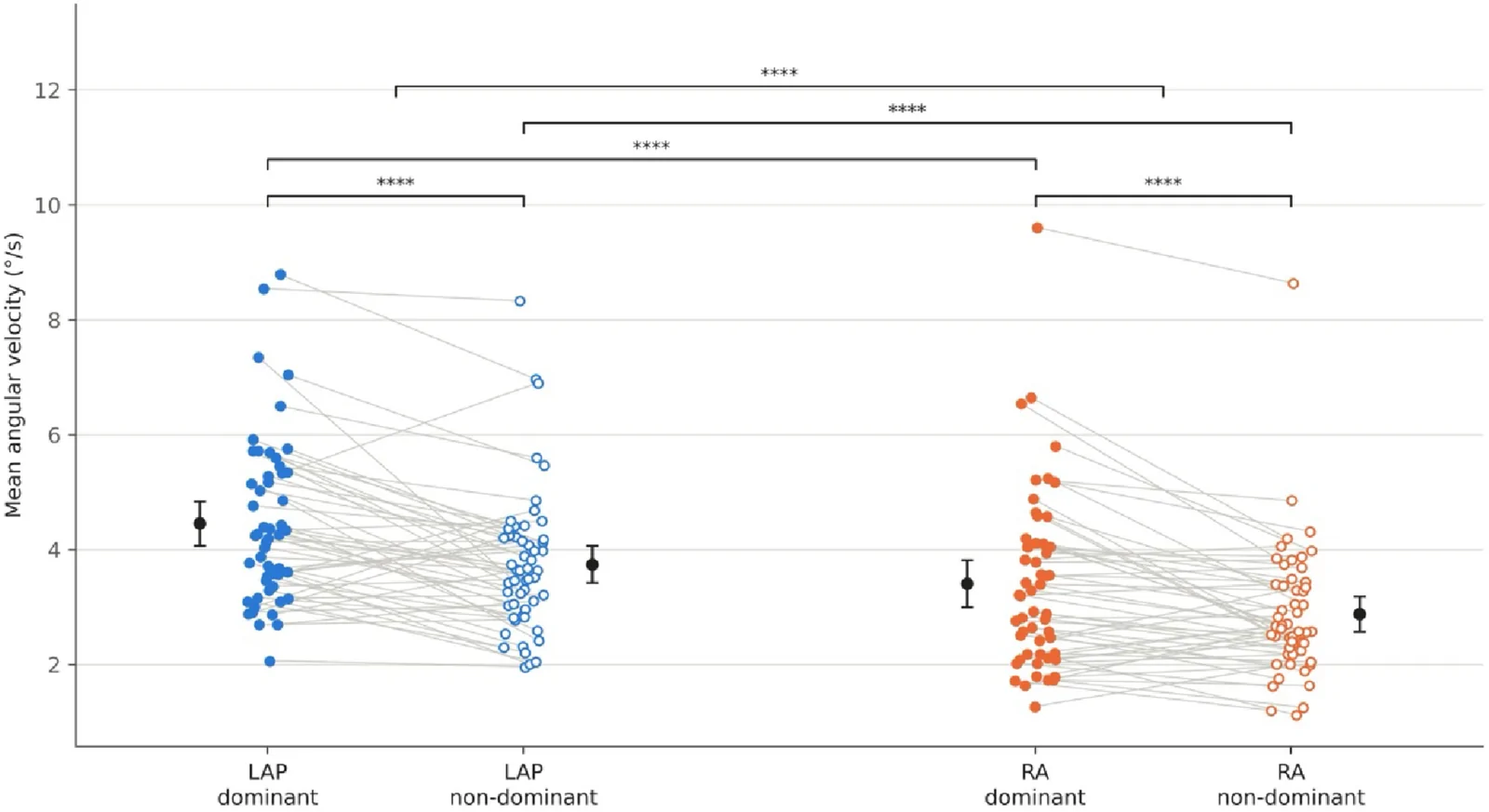
Angular velocity of the dominant and non-dominant upper arm for the three tasks combined, laparoscopic (LAP) and robotic platforms (RA); paired t-tests on participant-level means (***p < 0.001; ****p < 0.0001).

**Fig. 3:**
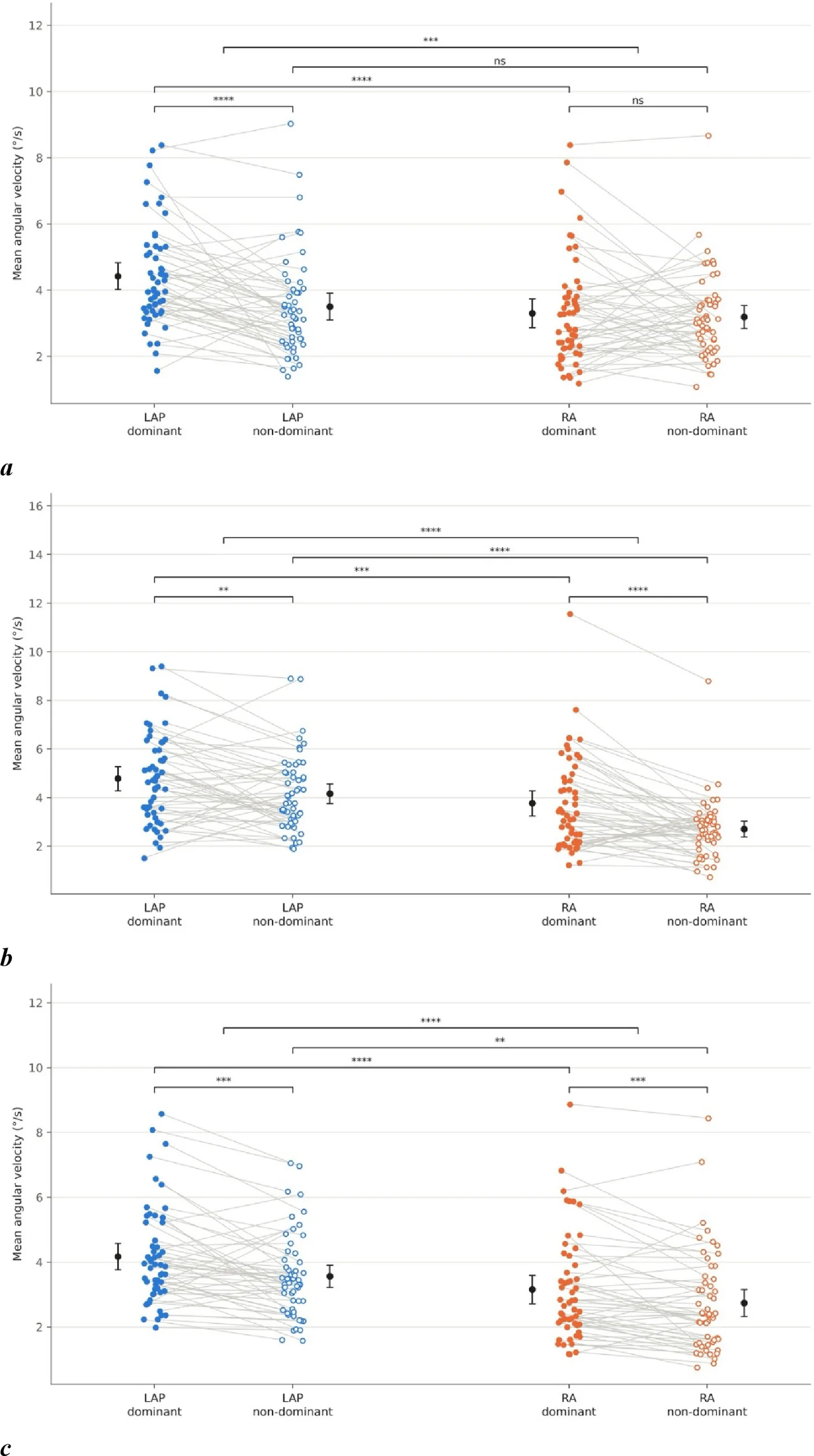
**a** Task 1 placing rings on spikes. **b** Task 2 stacking cubes. **c** Task 3 moving a hoop along a fixed twisted wire. Angular velocity (degrees per second) of the dominant and non-dominant upper arm during LAP and RA tasks; paired t-tests on participant-level means between arms, between modalities for each arm, and between modalities with both arms averaged (top bracket). ns, not significant; **p < 0.01; ***p < 0.001; ****p < 0.0001.

Paired t-tests on participant-level means demonstrated a statistically significant difference in angular velocity, jerk, and accelerations across tasks when comparing LAP versus RA (Figs. 2 and 3).

Significantly higher kinematic values were recorded during LAP compared to RA (*p* < 0.0001 to < 0.01). RA was associated with significantly lower jerk and angular velocity compared to LAP tasks (*p* < 0.0001 to < 0.01). The only exception was the non-dominant arm in Task 1 (*p* = 0.196; Fig. 3a). Overall, the trunk movement was less in RA tasks compared to LAP (*p* < 0.0001). Starting modality and task order were not associated with angular velocity or jerk (all *p* > 0.4); however, for jerk the LAP-RA difference was larger in participants who started with LAP (0.18 m/s³; 95% CI 0.03 to 0.33), consistent with a learning effect.

#### Analysis of tasks and hand dominance

Angular velocity was higher for the dominant than for the non-dominant arm in both LAP and RA (*p* < 0.001; Fig. 2), and in every task except Task 1 during RA (*p* = 0.547; Fig. 3).

#### Relationship between anthropometry and kinematics

**Fig. 4:**
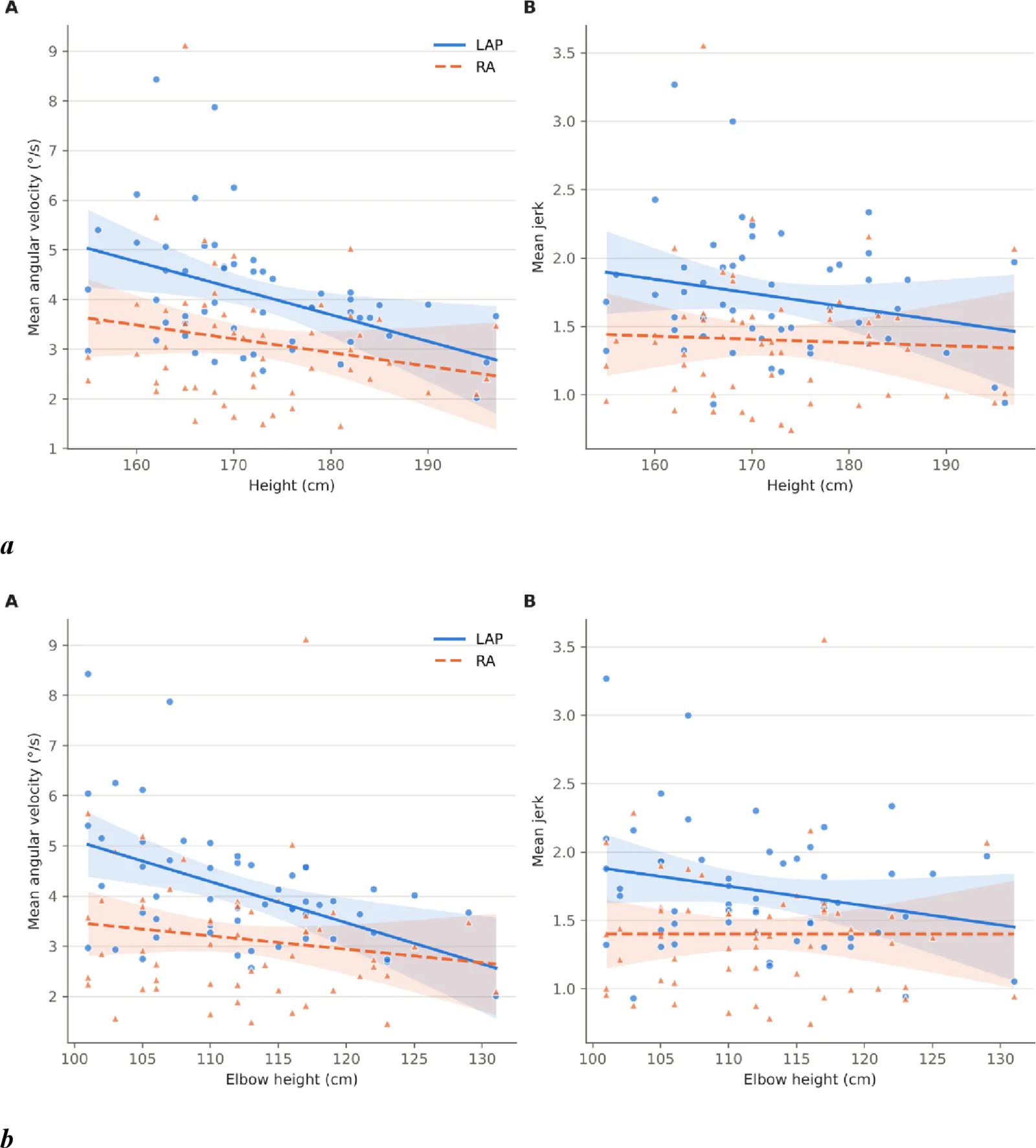
**a** Relationship between height and kinematics. Mean angular velocity (A) and mean jerk (B) against height during LAP and RA. Points are participant means; lines and shaded bands are model-predicted values with 95% confidence intervals from the linear mixed-effects model. **b** Relationship between elbow height and kinematics. Mean angular velocity (A) and mean jerk (B) against elbow height during LAP and RA. Points are participant means; lines and shaded bands are model-predicted values with 95% confidence intervals from the linear mixed-effects model.

The association between anthropometry and kinematics depended on the surgical modality (Table 1). Linear Mixed-Effects Models (controlling for participant random effects and sequence/period order) revealed a significant Anthropometry × Modality interaction for elbow height (angular velocity: −0.55 °/s per 10 cm, 95% CI − 0.83 to − 0.28, *p* < 0.001; jerk: −0.142 m/s³ per 10 cm, 95% CI − 0.240 to − 0.044, *p* = 0.005), which remained significant after Holm correction and in all sensitivity analyses. The interactions for height (angular velocity *p* = 0.015; jerk *p* = 0.034) did not remain significant after correction (*p* = 0.076 and *p* = 0.135), and arm span showed no interaction (*p* ≥ 0.12). Specifically, a significant inverse relationship was observed in LAP tasks, where decreasing elbow height was associated with greater angular velocity (− 0.82 °/s per 10 cm; 95% CI − 1.34 to − 0.31; *p* = 0.002) (Fig. 4). Conversely, these anthropometric features were not significantly associated with kinematics during RA tasks. Although no statistical significance was identified between sex and kinematics after accounting for anthropometry, the participants at the lower end of the anthropometric spectrum predominantly consisted of females, whilst the male participants were at the upper end (Fig. 1).

**Table 1:** Linear mixed-effects model estimates for the upper-arm sensors (54 participants, 643 observations). Slopes are the association between each trait and the outcome within each modality; the interaction is the difference between the LAP and RA slopes. Models were adjusted for sex, task, task order, starting modality and arm, with random intercepts for participant and task bout. Bold: *p* < 0.05.Holm p: adjusted across the eight interaction tests

Outcome	Trait (unit)	Slope in LAP (95% CI)	Slope in RA (95% CI)	Interaction (95% CI)	*p*	Holm *p*
Angular velocity (°/s)	Height (per 10 cm)	−0.53 (− 0.95 to − 0.11)	−0.28 (− 0.70 to 0.14)	−0.26 (− 0.47 to − 0.05)	**0.015**	0.076
Angular velocity (°/s)	Elbow height (per 10 cm)	−0.82 (− 1.34 to − 0.31)	−0.27 (− 0.78 to 0.25)	−0.55 (− 0.83 to − 0.28)	**< 0.001**	**< 0.001**
Angular velocity (°/s)	Arm span (per 10 cm)	−0.19 (− 0.55 to 0.17)	−0.05 (− 0.41 to 0.32)	−0.14 (− 0.32 to 0.04)	0.120	0.241
Angular velocity (°/s)	Hand size (per 1 cm)	−0.02 (− 0.29 to 0.25)	0.18 (− 0.10 to 0.45)	−0.19 (− 0.34 to − 0.05)	**0.008**	**0.0499**
Jerk (m/s³)	Height (per 10 cm)	−0.103 (− 0.265 to 0.060)	−0.023 (− 0.186 to 0.139)	−0.079 (− 0.152 to − 0.006)	**0.034**	0.135
Jerk (m/s³)	Elbow height (per 10 cm)	−0.142 (− 0.342 to 0.058)	0.000 (− 0.201 to 0.200)	−0.142 (− 0.240 to − 0.044)	**0.005**	**0.032**
Jerk (m/s³)	Arm span (per 10 cm)	0.021 (− 0.116 to 0.158)	0.040 (− 0.097 to 0.177)	−0.019 (− 0.082 to 0.044)	0.553	0.553
Jerk (m/s³)	Hand size (per 1 cm)	0.042 (− 0.059 to 0.142)	0.093 (− 0.007 to 0.194)	−0.051 (− 0.102 to − 0.001)	**0.048**	0.143

#### Hand size

**Fig. 5:**
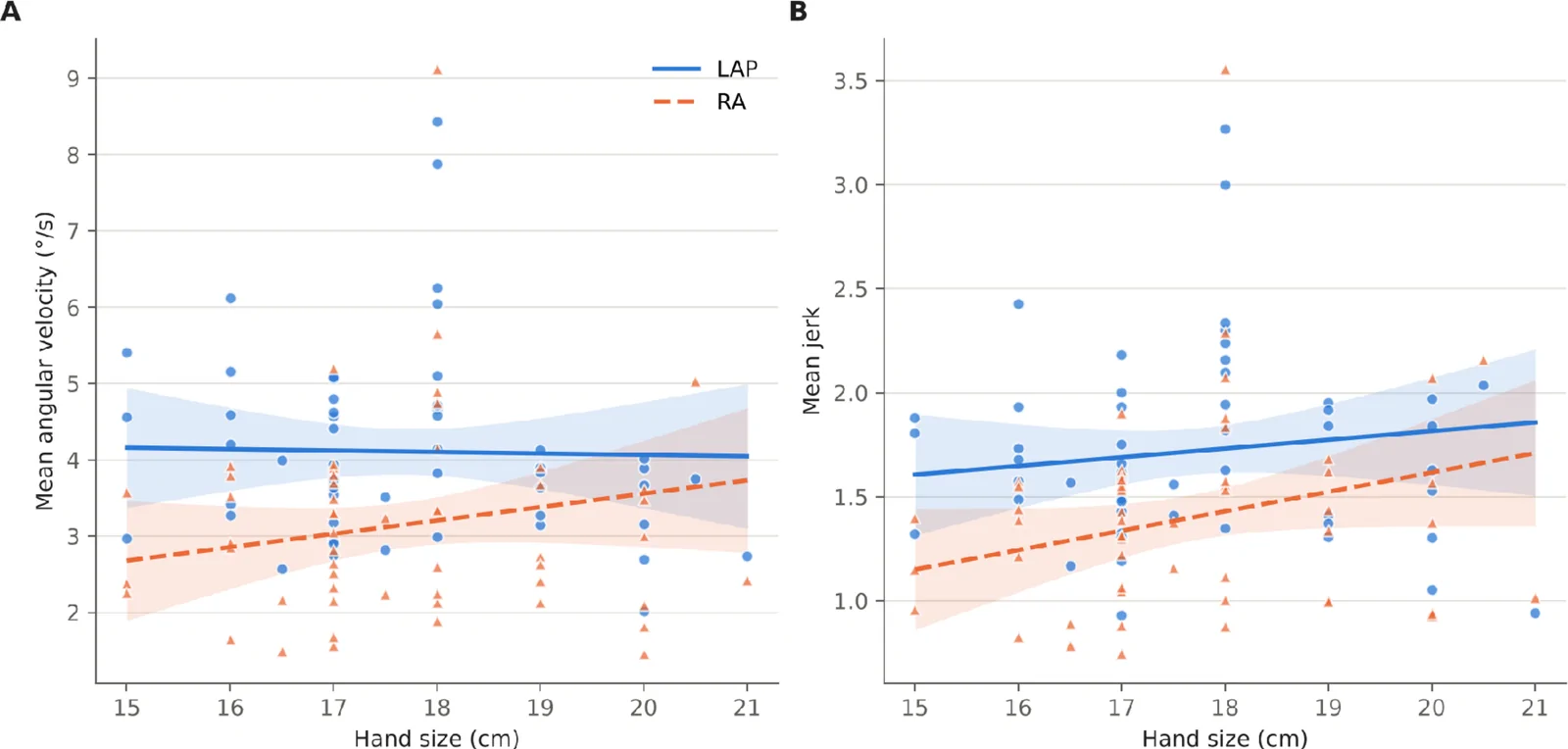
Relationship between hand size and kinematics. Mean angular velocity (A) and mean jerk (B) against hand size during LAP and RA. Points are participant means; lines and shaded bands are model-predicted values with 95% confidence intervals from the linear mixed-effects model.

Hand size interacted with modality for angular velocity (*p* = 0.008; Holm-adjusted *p* = 0.0499), but this reflected a non-significant positive slope during RA rather than an association during LAP (*p* = 0.892) (Fig. 5).

## Discussion

This study objectively investigated surgical motion across the two current MIS platforms, to provide a deeper understanding of the interaction between anthropometry, operative modality and surgical motion. The understanding of surgical motion in the context of variable surgeon build will provide greater insight into exploring the downstream consequences of cumulative physical strain and future WMSI risk. The key finding is an inverse relationship between upper body kinematics and anthropometry. Surgical novices with lower elbow height exhibited greater angular velocity during LAP, but not RA, tasks; the corresponding association with height was weaker. This significant difference in motion reflects not only the influence of anthropometry but also lends insight into the interaction between the user and the simulated minimally invasive platform i.e. motion is greater for the same tasks in LAP than in RA. In addition, we have demonstrated that in surgical novices the dominant hand is preferred regardless of modality.

WMSI amongst surgeons is a growing concern and incorporating ergonomic training early in a surgeon’s career is being recognised as an opportunity to discourage the adoption of harmful postures and techniques that could increase an individual’s WMSI risk. The physical impact of performing LAP has long been highlighted as a risk factor for WMSI, particularly when operating on patients with a high BMI or performing lengthy, technically complex procedures [8] whereas surgeon ergonomics has been touted as one of the advantages of the RA approach [19]. RA is not without ergonomic issues, with neutral, or ergonomic, postures at the da Vinci console only being achieved for individuals within the height range of 155–200 cm, as the viewing angle is limited to 40–60 degrees, and can result in neck and trapezius muscle region pain for those outside of this height range [20]. In the LAP setting, the optimum operating table height is 20–30% lower than the lead operator’s elbow height, as this enables the surgeon to maintain neutral postures during the procedure. Specialised operating tables can enable the surgeon to work at a lower height, 635 mm versus 720 mm [21], however, the addition of the patient’s abdominal thickness does need to be considered [6], particularly in the case of patient obesity. As a result, many surgeons find it challenging to work with an optimal ergonomic setup [6, 22, 23]. A key strength of our study findings is highlighting the interaction between intrinsic anthropometry and workstation adaptability. In the LAP setting, the operating table was fixed at a standard 72 cm height, whereas the RA console was height-adjustable. Consequently, the observed kinematic differences may reflect an anthropometry-by-interface interaction, suggesting that fixed-height setups may require greater kinematic compensation from operators with lower elbow height, whereas adjustable consoles may reduce this disparity. However, LAP and RA also differ in posture (standing versus seated) and instrument design, and the present design cannot separate these factors from workstation adjustability.

Jerkiness is a measure that implies reduced control in movement and quantitatively demonstrates a sudden change in motion [24], hence, increased jerk indicates more abrupt changes in motion, for example in response to the resistance from operating tools [25]. Raised patient BMI can also contribute to increased mean jerk and angular velocity of the upper extremities of the body, particularly the head, trunk and upper arms, regardless of whether the operator is a novice or expert surgeon [25]. In our study, the higher angular velocity values, especially in LAP tasks indicate faster rotation of the upper arms to manipulate the instruments and perform the desired surgical manoeuvre compared to RA tasks. As tasks were time-fixed and task performance was not recorded, this cannot be interpreted as lower efficiency. Further studies are needed to explore the real-world impact of additional variables, such as raised patient BMI or poor ergonomic setup, on kinematic values in LAP and RA settings to explore the association with WMSI risk.

Although simulated tasks lack real-world applicability, they play an important role in skill acquisition and have been incorporated into surgical training programmes to move novices along the learning curve, prior to performing surgery on patients. Within the live surgical environment, factors could contribute to bias, including theatre set-up, background noise, and patient factors, therefore making it more difficult to determine the relationship between anthropometry and motion [26]. Novices have been reported to have greater or more exaggerated upper body movements [25, 27] when performing simulated tasks compared to their expert counterparts, as they lack the economy of movement gained from years of experience [25], however, their posture is reported to be better as they have not had time to develop coping habits or strategies [28]. The focus on surgical novices in this study was chosen to enable the capture of movements without the influence of compensatory techniques gained from exposure to surgical practice, thereby reducing bias. However, novices also show greater and more variable movement because of unfamiliarity and early motor learning, and the findings may not apply to experienced surgeons. The ISSUE study is investigating the impact of surgery on the surgeon in a live surgical setting across three surgical modalities (open/laparoscopic/robotic) and includes kinematic analysis to explore whether our findings from the simulated setting are confirmed in a population of experienced surgeons [29].

The link between surgeon anthropometry and surgical motion and future WMSI is currently speculative however, self-reported data from surgeon surveys does support an association [14]. Ergonomic training, strengthening exercises and behavioural changes have shown substantial improvement in other settings [30–32], and lessons could be learned from sports science, training to prevent injury as well as for performance, through targeted physiotherapy programmes [33, 34]. Mitigating future WMSI risk is therefore important in maintaining the long-term health of the surgical workforce and could be incorporated early in surgeons’ careers to optimise their kinematics [8, 25]. Currently, there is no standardised guidance available to support the achievement of this aim, although the role of artificial intelligence (AI) and prediction modelling could be used to inform and develop ergonomic guidelines and public health policies to protect the surgical workforce [28], as well as individualising theatre set-up in order to create the optimum working environment.

### Limitations

Every effort was made to minimise bias by using identical tasks across both surgical platforms and avoiding robot-assisted (RA) simulated exercises. Although some participants may have had prior exposure to laparoscopic (LAP) or RA simulation, neither are included in the medical school curriculum and any previous experience is likely to have been limited. Modality order and task order were included as covariates, but a carry-over effect was observed for jerk, and residual learning effects cannot be excluded. Task order was not formally randomised, and testing day and starting modality were linked because participants self-selected their testing day; however, participants were unaware of their assigned starting modality until commencement of testing. Table height was fixed for LAP but adjustable on request at the RA console, which may have influenced surgical motion, although this reflects real-world practice where RA systems offer greater ergonomic customisation. Several traits and outcomes were analysed; Holm correction was applied to the primary interaction tests, but secondary results should be interpreted with caution. Furthermore, as participants were medical students, prior exposure to video gaming or sports was not formally quantified and remains an unmeasured potential confounder. Extrapolation of these simulated findings to experienced, practising surgeons must be appropriately qualified.

## Conclusions

This study gives new insights into the inverse relationship between anthropometry, in particular elbow height, and upper-arm kinematics during simulated LAP, compared to RA, tasks. These findings may help to generate hypotheses about the patterns of WMSIs reported by surgeons; however, longitudinal real-world studies are needed to confirm these associations. Incorporating surgeon specific ergonomic training early in surgical careers, as well as designing training interventions that increase understanding of surgeon ergonomics, may contribute to a holistic approach to reducing future WMSI.

## Data Availability

Due to ethical constraints the data will not be available however will be stored in the University of Leicester data repository.
